# Alterations in Small Non-coding MicroRNAs (miRNAs) and the Potential Role in the Development of Aseptic Loosening After Total Hip Replacement: Study Protocol for an Observational, Cross-Sectional Study

**DOI:** 10.7759/cureus.72179

**Published:** 2024-10-23

**Authors:** Spyridon Papagiannis, Irini Tatani, George Kyriakopoulos, Zinon Kokkalis, Panagiotis Megas, Constantinos Stathopoulos, Katerina Grafanaki, John Lakoumentas

**Affiliations:** 1 Orthopedics and Traumatology, University General Hospital of Patras, Patras, GRC; 2 Department of Biochemistry, School of Medicine, University of Patras, Patras, GRC; 3 Department of Dermatology, School of Medicine, University of Patras, Patras, GRC; 4 Department of Medical Physics, School of Medicine, University of Patras, Patras, GRC

**Keywords:** aseptic loosening, mirnas, non-coding rnas, periprosthetic osteolysis, revision, total hip arthroplasty

## Abstract

Introduction: Total hip arthroplasty (THA) is one of the most successful and effective surgeries for the treatment of hip osteoarthritis, with good rates in terms of survival, pain relief, and patient functional recovery. Aseptic loosening (AL) accompanied by periprosthetic osteolysis (PPOL) is the most frequent late complication, accounting for almost 50% of all revision surgeries. The primary purpose of this observational, cross-sectional study is to identify alterations in small, non-coding RNAs, miRNAs, that could be involved in the pathogenesis of AL and PPOL following THA.

Methods/design: Sixty-three patients will be included in this study and will be divided into three groups (21 in each group): Group A (control group), including patients undergoing primary THA due to degenerative hip osteoarthritis, Group B including patients without clinical and radiological evidence of PPOL/AL following primary THA, and Group C including patients with clinical and radiological evidence of PPOL and AL undergoing revision surgery following primary THA. Blood samples will be collected from all patients. Peripheral blood samples from Group A and C patients will be collected prior to surgery while synovial membrane samples will also be collected intraoperatively. Synovial membrane samples will not be collected from Group B patients since they are not candidates for any surgical intervention. The relative expression of miRNAs let-7i-5p, let-7e-5p, miR-15a-5p, miR-30a-3p, and miR-130a-3p, will be measured using real-time quantitative PCR (qRT-PCR) at baseline from all patients.

Conclusion: The primary goal is to identify the expression of inflammation-related miRNAs that could play a role in the pathophysiology of AL and highlight the differences among patients with confirmed AL, patients with degenerative hip disease, and patients with no signs of AL following THA. The secondary goal is to use these miRNAs as biomarkers to estimate the possibility for a patient to develop AL after total hip replacement, and also as possible treatment targets.

Our study has been registered with an International Standard Randomized Controlled Trial Number ID: ISRCTN25839413.

## Introduction

Total hip arthroplasty (THA) is one of the most effective surgical treatments in orthopedics. THA is implemented to treat severe hip osteoarthritis, aiming to reduce pain and enhance hip joint function. Survivorship of THA implants has progressively improved, such that approximately 80% of implants function optimally 25 years post-operatively [1]. Advances in materials, implant designs, and surgical techniques have decreased the incidence of complications; however aseptic loosening (AL), the clinical end stage of periprosthetic osteolysis (PPOL) remains the most frequent complication, accounting for at least 50% of all revision surgeries according to national registries [2]. Understanding the pathophysiology of PPOL and AL is of paramount significance in improving THA outcomes. There is strong evidence that AL occurs secondary to a chronic, low-grade, inflammatory response that leads to a remodeling imbalance of the bone-implant interface. This low-grade inflammation represents an adverse host response to prosthetic particles and byproducts [3]. Wear debris are formed at bearing surfaces, modular interfaces, and bone-implant interfaces. The inflammatory response to wear particles depends on the size, shape, composition, and number of particles. Particles sized between 0.1 and 1 μm seem to have the highest inflammatory effect. In terms of origin, polyethylene, polymethylmethacrylate (PMMA) and metal byproducts are the most inflammatory ones [4].

Prosthetic byproducts interact with innate immunity receptors located on the surface of immune cells. These receptors activate different intracellular pathways and result in the upregulation and release of inflammatory cytokines, chemokines, and reactive oxygen species (ROS). Tumor necrosis factor-alpha (TNF-a), interleukins (IL)-1, 6, 17, interferon-gamma (IF-γ), colony-stimulating factors (M-CSF and GM-CSF), prostaglandins (PGE2) and matrix metalloproteinases are considered potent contributors in particle-induced osteolysis [5,6]. The accumulation of wear particles is also associated with a marked increase in the expression of receptor activator NF-kB ligand (RANKL) and its receptor, RANK. It is well established that the activation of the RANKL/RANK complex leads to the differentiation of osteoclast precursors and activation of mature osteoclasts, resulting in bone resorption. TNF can also greatly enhance the expression and activity of RANKL. The RANK-RANKL pathway seems to be a key regulator of osteoclast-induced bone resorption, while recently published literature reported the potential role of microRNAs in RANKL-induced osteoclastogenesis [7-10].

MicroRNAs (miRNAs) are small, single-stranded, non-coding RNA molecules (containing about 22 nucleotides) that function in RNA silencing and post-transcriptional regulation of gene expression. miRNAs function through base-pairing with complementary sequences within mRNA molecules. As a result, these mRNAs are silenced either by cleavage of the mRNA strand into two pieces or by translational repression of the mRNA [11-13]. miRNAs are abundant in many cell types, while different studies demonstrated the presence of extracellular circulating miRNAs [14]. Extracellular circulating miRNAs are most commonly connected with Ago-2 proteins and identified in blood samples as stable free protein complexes, while some studies described miRNA complexes with high-density lipoproteins (HDL), and miRNAs stored in exosomes. Extracellular circulating miRNAs have the ability to remain stable in blood circulation by being encapsulated in vesicles and in that way protected from circulating RNases. Ago-proteins also help to protect miRNAs from enzymatic cleavage, while studies indicated that miRNAs could remain stable for 1 year if stored at −70°C. The aforementioned biochemical behavior and stability of miRNAs can make them useful biomarkers.

## Materials and methods

Study design

**Figure 1:**
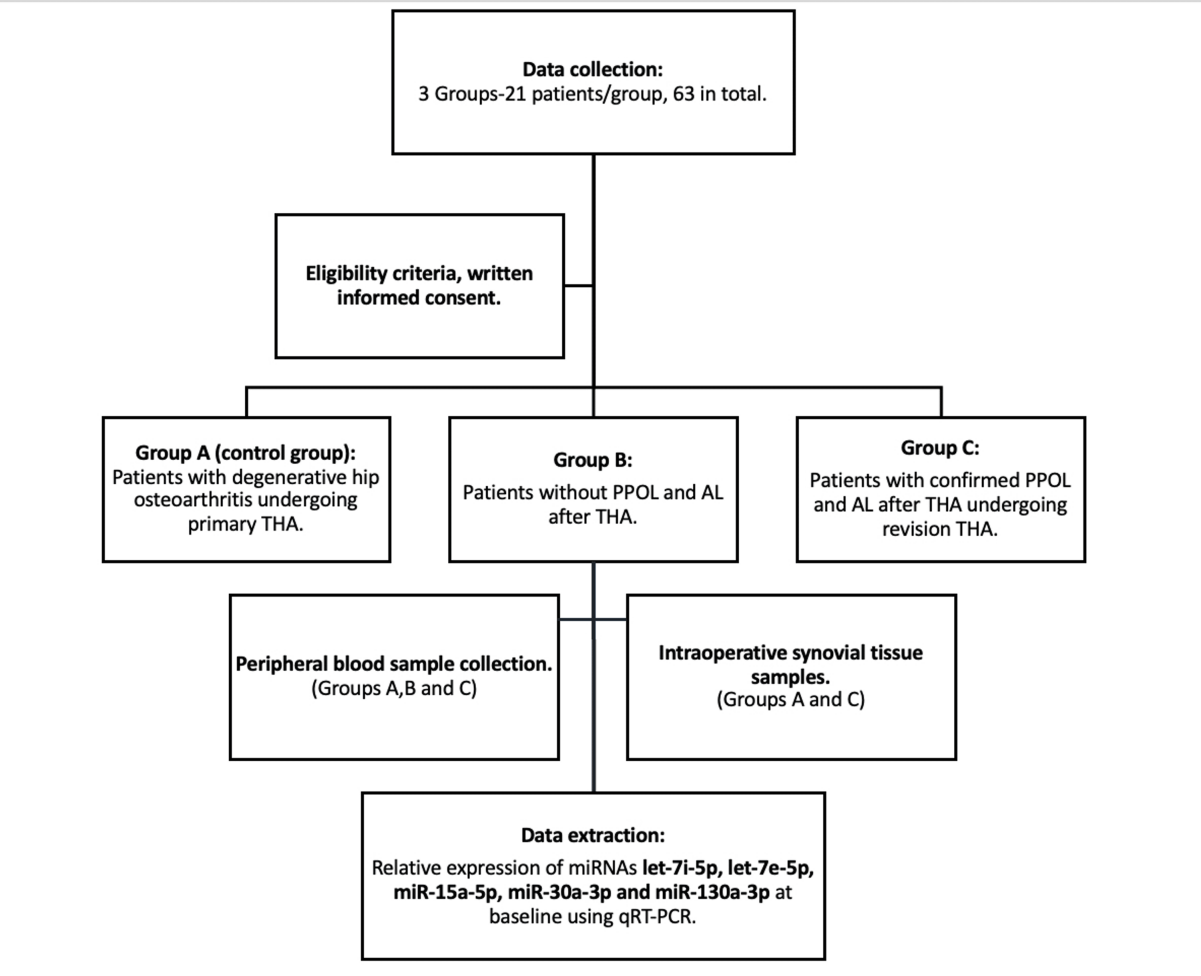
Study flowchart THA: total hip arthroplasty; PPOL: periprosthetic osteolysis; AL: aseptic loosening

Our study is a cross-sectional, observational study. Patients enrolled in this study will be divided into three groups. Group A(control group): Patients with clinical and radiological signs of degenerative hip osteoarthritis undergoing primary THA, Group B: Patients with no clinical and radiological findings of PPOL and AL following primary THA for hip osteoarthritis, and Group C: Patients with clinical and radiological evidence of PPOL and AL undergoing revision surgery following primary THA for hip osteoarthritis. A flowchart of our study is presented in Figure 1.

Statistical analysis

These groups will be available for analysis, and a set of continuous measurements will be compared against them, along with a small set of demographic variables. Statistical significance regarding the miRNA set of continuous measurements will be assessed. Since miRNA variables will be estimated once, one-way analysis of variance (ANOVA) will be applied for the detection of differences. Pairwise post-hoc comparisons will be held via the Bonferroni or Tukey test. Since our three groups imply a logical ordering, one may divide the patients into two groups, by merging neighboring groups: for example, A-B versus C, or A versus B-C. The latter scenario is useful in the sense that one may employ receiver-operating characteristic (ROC) curve analysis to identify optimal cutoffs of the candidate miRNA biomarkers. Finally, multiple candidate biomarkers will be used to predict any potential 2-class variable (as obtained by merging two groups), using the traditional logistic regression method. The statistical significance threshold will be set at 5% and the only described hypothesis testing procedure will be ANOVA, on which our power analysis will be based. Using the R statistical language along with the RStudio software interface, a minimum amount of 51 patients (17 per group) should be enrolled to assume that the differences among the groups will be strongly considerable.

Eligibility criteria

The inclusion criteria for our study are the following: male and female patients above 40 years of age, patients undergoing primary THA due to degenerative hip osteoarthritis (Group A), patients with primary THA, without clinical and radiological evidence of PPOL/AL (Group B), and patients with clinical and radiological evidence of PPOL and AL undergoing revision surgery following primary THA (Group C). Exclusion criteria involve patients with revision THA surgery, patients with bilateral total hip replacement or hemiarthroplasty on the contra-lateral hip, pre-existing other joint replacement, positive inflammatory markers, and intraoperative cultures indicating potential septic loosening, patients suffering from cancer or immunodeficiency, patients receiving chemotherapy or immunosuppressive medication, patients suffering from autoimmune diseases, severe cognitive defects or psychiatric disorders and inability to provide written informed consent to the study.

Variables

A thorough medical history will be recorded for all eligible patients. The exclusion or confirmation of AL for Group B and Group C patients respectively, will be made based on clinical examination and radiological findings from the anteroposterior and lateral hip radiographs. Patients’ clinical and functional levels will be assessed and documented according to the Harris Hip Score and SF-36 Score. Radiolucencies among bone-implant interface, cup migration or tilting, varus tilting and/or gross subsidence of the femoral stem, and eccentric position of the femoral head in the acetabular cup indicating wearing-out of the polyethylene are all considered radiological signs of implant loosening and will be thoroughly examined in the enrollment phase [15]. Other causes of functional limitations and pain after THA such as periprosthetic fractures, dislocations, implant malposition, or failure will be ruled out for Group C patients based on clinical and radiological findings. Septic loosening will also be ruled out using blood tests (Total White Blood Count) and serology tests including erythrocyte sedimentation rate (ESR) and C-reactive protein (CRP). Intraoperative cultures will be obtained to confirm the aseptic environment of implant loosening. Blood samples will be collected from all patients. Peripheral blood samples from Groups A and C patients will be collected prior to surgery while synovial membrane samples will also be collected intraoperatively. Synovial membrane samples will not be collected from Group B patients since they are not candidates for any surgical intervention.

Setting

Patients’ clinical examination, radiographic evaluation, written informed consent, enrollment, and sample collection will be performed at the Orthopedic Department of our University Hospital. Data analysis and extraction will be performed at the Biochemistry Department.

miRNA expression analysis

Initially, the serum will be centrifugated for 10 minutes at 2000 rpm and the supernatant will be collected and stored at -70°C. RNAs will be isolated from the samples using an enrichment procedure with small RNAs, while the absorbance of the samples will be measured in the ultraviolet spectrum, specifically at 260 nm, to calculate the amount of RNAs present in each sample. All RNA molecules from our samples will then be polyadenylated using the polyadenylation enzyme Poly(A). In order to avoid any possible sample contamination with proteins, pure RNA solution will be recovered. For this purpose, samples will be extracted using phenol to denature the proteins, and then with chloroform to remove residual phenol. Finally, precipitation with ethanol and NaOAc (100%) will be performed to isolate RNAs in precipitate form. After the isolation of pure RNAs, the corresponding cDNA will be synthesized using the reverse transcriptase enzyme according to the BioScriptTM Reverse TranscriptaseTM protocol. miRNAs expression levels in the samples will be detected using the standard two-step real-time PCR protocol.

The relative expression of five miRNAs, let-7i-5p, let-7e-5p, miR-15a-5p, miR-30a-3p, and miR-130a-3p, will be measured at baseline from all patients enrolled in our study. According to current published literature, these miRNAs have been associated with the expression of peri-inflammatory cytokines at the post-transcription level and play fundamental roles in inflammatory and autoimmune processes involved in various diseases such as ankylosing spondylitis [16], diabetes [17], systemic sclerosis, rheumatoid arthritis [18], and various tumors [19]. In addition to these miRNAs, an miRNA reference (normalizer), miRNA RNU6, will also be measured in all samples. For each of these five miRNAs under investigation, a separate reagent mixture will be prepared, and a duplicate reaction will be performed to obtain a more accurate measurement and minimize the possibility of experimental error. The analysis of the qRT-PCR results will be performed using the 2-ΔΔCT method. The threshold cycle (CT) is the cycle at which the fluorescence level reaches a certain amount. This method directly uses the CT information generated from a qPCR system to calculate relative gene expression in target and reference samples, using an miRNA reference as the normalizer. The ΔΔCT value is calculated from the equation ΔΔCT = (CT target - CT RNU6) - (CT avg ctr - CT avg RNU6 ctr). CT target is the CT value of the tested miRNA in each sample, CT RNU6 is the CT value of the reference miRNA (RNU6) in each sample, CT avg ctr is the average CT value of the tested miRNA control samples, and CT avg RNU6 ctr is the average CT value of the reference miRNA (RNU6) in all control samples.

## Results

This is an ongoing trial with results expected to be obtained within the next 4 months, following the completion of data analysis. Data collection has been completed 3 months ago, while our data analysis phase is already in progress.

The primary goal of this trial is to evaluate the expression of five specific, inflammation-related miRNAs, let-7i-5p, let-7e-5p, miR-15a-5p, miR-30a-3p, and miR-130a-3p, in patients with AL following THA and their potential role on the ongoing inflammatory process of PPOL. The rationale behind this research stems from the significant role of miRNAs in the post-transcription regulation of various inflammatory processes, as reported in recently published literature. However, no data directly associating specific mi-RNAs with the development of PPOL is available.

Our hypothesis includes different expressions of these miRNAs between patients with confirmed AL and asymptomatic patients following THA, indicating either the protective or inductive role of these miRNAs on the inflammatory procedure of AL. Possible outcomes may reveal circulating biomarkers in peripheral blood that could help us estimate the possibility of a patient developing AL following THA. In the long term, these miRNAs could be also used as possible targets for the development of new and more effective pharmaceutical treatments or even gene therapies.

## Discussion

Implant-related inflammation is the key process in the development of AL and consists of two phases: a foreign body reaction that begins immediately after implantation, and a debris-induced reaction that occurs years after implantation, due to immune recognition of micron-sized wear particles. Particle debris triggers an immunological response through the activation of monocytes, macrophages, and dendritic cells, which leads to the upregulation of pro-inflammatory cytokines, macrophage receptors with collagenous structure (MARCO), and nitric oxide (NO) [20]. Macrophages are considered the primary cell type involved in host-implant integration due to their significant phenotypic and functional variability [21]. Activated, pro-inflammatory macrophages, known as M1 macrophages, express matrix-remodeling enzymes, secret RANKL and M-CSF, and contribute to the differentiation of multinucleated osteoclasts, which stimulate bone resorption. Conversely, alternatively activated, or anti-inflammatory macrophages, referred to as M2 macrophages, release relatively modest amounts of pro-inflammatory cytokines and generate substantial concentrations of the powerful regulatory cytokine IL-10. M2 macrophages are mostly thought to act as mediators of wound healing and seem to express high amounts of scavenger, mannose, and galactose receptors. Additionally, polymers used as bearing-surface materials in THA implants can produce short carbon chains that bind directly to toll-like receptor 2 (TLR2) on monocytes and macrophages which triggers NF-κB activation and further transcription of pro-inflammatory cytokines [22]. Apart from TLR2 activation, implant debris also stimulate “endosomal destabilization” and the release of cathepsins into the cytosol of activated macrophages, promoting the assembly of NALP3 inflammasome [23]. The NALP3 inflammasome is a multi-protein complex that activates caspase one, which then cleaves precursor molecules pro-IL-1β and pro-IL-18 into the active cytokines that contribute to the initiation of osteolysis. The process of osteolysis involves active resorption of bone matrix, while nearby macrophages also promote bone demineralization through the secretion of metalloproteinases, collagenases, and tissue-processing enzymes. Decreased bone-contacting surface areas due to bone demineralization promotes further implant loosening and wear-debris formation highlighting the fact that implant destabilization and bone demineralization are two strongly correlated phenomena that amplify each other.

To date, the exact role of miRNAs in the molecular pathways of PPOL remains uncertain. MicroRNAs seem to play a role in the regulation of macrophage polarization. Graff et Al. reported that M1 macrophages showed markedly up-regulated expression of miR-27a, miR-29b, miR-125a, miR-146a, and miR-155, whereas M2 cells expressed higher levels of miR-26a and miR-193b [24]. TLR and inflammasome/NLR signaling pathways also seem to be regulated by different microRNAs. miR-101 inhibits TLR2/TLR4-induced production of pro-inflammatory cytokines by deactivating the MAPK enzyme and is considered a positive regulator of innate immune responses [25]. In contrast, MiR-146a inhibits NF-κB activation and reduces the generation of pro-inflammatory cytokines in response to TLR ligation by targeting the signaling molecules TRAF6 and IRAK1 [26]. Few studies have investigated the role of miRNAs in the regulation of NALP3 inflammasome. According to Bauernfeind et al. [27], miR-223 expression can decrease NLRP3 expression and inhibit inflammasome activity by lowering IL-1β. Bandyopadhyay et al. reported that miR-133a-1 overexpression suppresses the activation of NLRP3, increases caspase one activity and enhances the release of mature IL-1β [28]. miRNAs also seem to be involved in the regulation of various inflammatory responses. Several miRNAs, including miR-106a, miR-4661, miR-98, miR-27, let7, and miR-1423p-5p, have been found to directly regulate the post-transcriptional expression of IL-10 cytokine [29]. IL-10 promotes M2 phenotype macrophage polarization that remains stable and cannot be reversed even within a pro-inflammatory microenvironment.

The main limitation of our study is that patients with previous primary total hip replacement included in Groups B and C had implants in service with different bearing surfaces, including metal-on-polyethylene, ceramic-on-polyethylene, and ceramic-on-ceramic. Biotribological studies have shown that bearing surfaces have different wear resistance, with metal-on-polyethylene implants producing the biggest amount of wear debris [30]. As mentioned earlier, the origin, size, and amount of wear debris can have different effects on the induction and perpetuation of the inflammatory process of PPOL and AL. This could possibly affect the ongoing inflammatory process based on the pre-existing THA in service as well as the expression of the miRNAs under investigation. Further studies could be conducted in the future to compare the expression of these miRNAs among patients having THA implants with different bearing surfaces in service.

## Conclusions

The primary purpose of our study is to investigate the expression of miRNAs let-7i-5p, let-7e-5p, miR-15a-5p, miR-30a-3p, and miR-130a-3p in patients with AL following primary THA and compare these results with patients suffering from degenerative osteoarthritis and control patients that remain asymptomatic without evidence of AL following THA. Based on these results, either the protective or inductive role of these miRNAs in the pathogenesis of PPOL could also be investigated. The secondary goal is to address the use of these miRNAs as biomarkers to evaluate the possibility of a patient developing AL after THA. Finally, these miRNAs could be also used as possible targets for the development of new and more effective pharmaceutical treatments or even gene therapies.
